# Higher Rates of *Clostridium difficile* Infection among Smokers

**DOI:** 10.1371/journal.pone.0042091

**Published:** 2012-07-27

**Authors:** Mary A. M. Rogers, M. Todd Greene, Sanjay Saint, Carol E. Chenoweth, Preeti N. Malani, Itishree Trivedi, David M. Aronoff

**Affiliations:** 1 Department of Internal Medicine, University of Michigan, Ann Arbor, Michigan, United States of America; 2 Ann Arbor Veterans Affairs Medical Center, Ann Arbor, Michigan, United States of America; Charité, Campus Benjamin Franklin, Germany

## Abstract

**Objectives:**

Cigarette smoking has been shown to be related to inflammatory bowel disease. We investigated whether smoking affected the probability of developing *Clostridium difficile* infection (CDI).

**Methods:**

We conducted a longitudinal study of 16,781 older individuals from the nationally representative Health and Retirement Study. Data were linked to files from the Centers for Medicare and Medicaid Services.

**Results:**

Overall, the rate of CDI in older individuals was 220.6 per 100,000 person-years (95% CI 193.3, 248.0). Rates of CDI were 281.6/100,000 person-years in current smokers, 229.0/100,000 in former smokers and 189.1/100,000 person-years in never smokers. The odds of CDI were 33% greater in former smokers (95% CI: 8%, 65%) and 80% greater in current smokers (95% CI: 33%, 145%) when compared to never smokers. When the number of CDI-related visits was evaluated, current smokers had a 75% increased rate of CDI compared to never smokers (95% CI: 15%, 167%).

**Conclusions:**

Smoking is associated with developing a *Clostridium difficile* infection. Current smokers have the highest risk, followed by former smokers, when compared to rates of infection in never smokers.

## Introduction

*Clostridium difficile* is ubiquitous in the environment. It has been found in water, foods such as meats and vegetables, soil, pets, farm animals, and on various surfaces in healthcare facilities [1]–[3]. Although *C. difficile* is relatively common in the environment, *C. difficile* infection (CDI) often follows exposure to antibiotics and is a significant antibiotic-associated colitis in hospitals and skilled nursing facilities [4]. Antibiotic use is widespread both in healthcare settings and in the community, with 304 prescriptions per every 1,000 adult outpatients in the United States (US) [5]. Yet, most individuals receiving antibiotics do not develop CDI and most individuals who are colonized by *C. difficile* do not exhibit symptomatic infection [6]. Therefore, we hypothesized that there must be other precipitating factors – such as patient characteristics and/or transmission-related components – that may influence the risk of CDI.

Cigarette smoking is a known risk determinant for both bacterial and viral infections through physiologic changes to the respiratory tract and alterations in both cell- and humoral-mediated immune responses [7]. In addition, pathogenic microorganisms are abundant in cigarettes; *Clostridium* species have been found in ≥90% of cigarette samples tested [8]. Since the relationship between smoking and CDI has yet to be reported, we sought to investigate whether smoking affected the probability of developing CDI. This was of particular interest because prior research has indicated complex associations between tobacco use and other types of colitis. There is a positive association between cigarette smoking and the risk of Crohn’s disease, but there is a negative association between current cigarette smoking and ulcerative colitis [9]. Therefore, it was unclear whether smoking would have a negative or positive effect on the development of CDI.

**Table 1:** Rate of *Clostridium difficile* Infection in Medicare Beneficiaries in the United States 1991–2007, by Smoking Status.

			Non-Smoker		Former Smoker		Current Smoker	
Characteristics		n, sample	Rate a	95% CI	Rate a	95% CI	Rate a	95% CI
Gender	Men	7352	176.9	109.1, 244.7	187.0	141.0, 233.0	254.3	172.2, 336.4
	Women	9429	193.2	157.0, 229.3	288.1	218.1, 358.1	308.2	218.7, 397.7
Race	African-American	2232	216.4	105.0, 327.7	235.5	120.1, 351.0	337.7	170.7, 504.7
	Other	14549	184.9	150.4, 219.4	228.2	183.7, 272.7	269.6	198.1, 341.2
Ethnicity	Mexican-American	700	235.0	90.4, 379.6	253.2	104.6, 401.8	326.2	0.0, 776.2
	Other Hispanic	486	177.4	74.7, 280.1	346.4	37.9, 654.9	– b	– b
	Non-Hispanic	15595	188.0	152.6, 223.3	225.4	180.3, 270.6	284.9	214.7, 355.1
Body mass index	<25.0 kg/m^2^	6677	181.0	132.2, 229.9	245.4	169.8, 320.9	266.0	172.9, 359.1
	25.0–29.9 kg/m^2^	6850	195.6	149.2, 241.9	206.5	154.7, 258.3	303.3	196.5, 410.1
	≥30.0 kg/m^2^	3254	193.2	117.3, 269.1	253.3	162.9, 343.7	276.7	162.0, 391.3
Alcohol use	Yes	10368	168.4	129.3, 207.4	197.5	155.0, 240.0	236.9	165.2, 308.6
	No	6413	210.0	157.0, 262.9	301.3	209.0, 393.6	388.8	260.9, 516.6
Heart disease	Yes	3457	257.9	182.0, 333.8	254.1	161.1, 347.1	372.7	211.7, 533.6
	No	13324	173.4	137.1, 209.7	220.7	175.3, 266.1	262.8	201.2, 324.5
Diabetes mellitus	Yes	1999	283.0	171.0, 395.0	252.9	145.3, 360.6	368.5	158.5, 578.4
	No	14782	177.6	145.8, 209.4	225.5	182.3, 268.8	272.4	201.8, 343.1
Chronic lung disease	Yes	1178	334.2	73.4, 594.9	429.0	262.0, 596.0	424.0	186.2, 661.8
	No	15603	184.1	151.5, 216.8	211.3	172.0, 250.6	265.1	206.6, 323.6
Stroke	Yes	1083	345.1	118.3, 571.8	361.2	153.4, 569.0	472.3	164.0, 780.7
	No	15698	180.1	147.0, 213.2	219.4	176.0, 262.7	268.3	206.2, 330.4
Irritable bowel disease	Yes	1508	189.9	101.8, 278.0	303.1	168.6, 437.5	392.1	178.8, 605.3
	No	15273	189.0	152.6, 225.5	220.5	173.9, 267.1	272.2	209.0, 335.4
Ulcerative colitis	Yes	308	585.8	148.7, 1023.0	437.4	154.8, 720.1	781.0	17.5, 1544.4
	No	16473	182.0	148.6, 215.5	223.9	178.1, 269.8	271.6	209.7, 333.5
Overall		16781	189.1	156.1, 222.2	229.0	185.0, 273.0	281.6	218.9, 344.4

Footnotes:

a Number of individuals with *C. difficile*/100,000 person-years, survey-weighted to the reference population.

b Unstable rates due to small numbers.

## Methods

Data were from the longitudinal Health and Retirement Study which is a nationally-representative study of older Americans over the age of 50 years [10]. This ongoing study was designed to obtain information from older community-dwelling US residents via biennial interviews. A multi-stage area probability sample of households was utilized. Information was collected regarding retirement, employment, family structure, insurance, housing, finances, demographics, and physical and functional health. Data were linked to files from the Centers of Medicare and Medicaid Services (CMS), years 1991–2007 (n = 16,781 in sample). For purposes of this study, the following CMS files were used: Inpatient Standard Analytical Files (SAFs), Outpatient SAFs, Skilled Nursing Facility SAFs, Home Health Agency SAFs, Carrier (Part B) SAFs, and the Denominator files.

CDI was determined from a physician’s diagnosis of *C. difficile* as recorded in hospital, skilled nursing facility, emergency department, home health agency or clinic/outpatient files, as recorded by ICD-9-CM code 008.45 which is the only ICD-9-CM code for specifically identifying CDI. Initially, rates of CDI were calculated for the entire population (number of individuals with CDI per person-years of observation) and then, for subgroups based on patient characteristics. Subjects were classified by smoking status based on their answers to these questions, “Have you ever smoked cigarettes? By smoking we mean more than 100 cigarettes in your lifetime; do not include pipes or cigars. Do you smoke cigarettes now?” Subjects were classified into “never smokers” if they indicated that they never smoked cigarettes throughout their entire lives, “former smokers” if they smoked cigarettes prior to their entrance into the Health and Retirement Study, and “current smokers” if they smoked cigarettes during the study observation period (prior to the first diagnosis of CDI for those subjects with CDI).

**Table 2:** Association between Smoking Status and *Clostridium difficile* Infection in Medicare Beneficiaries in the United States, 1991–2007.

		*Clostridium difficile* infection			*Clostridium difficile*-related Visits		
Adjustment	Smoking Status	OR a	95% CI	p value	IRR b	95% CI	p value
Adjusted for age, sex, race:	Never smoker	1.00	(reference)		1.00	(reference)	
	Former smoker	1.38	1.11, 1.71	0.004	0.95	0.62, 1.47	0.826
	Current smoker	1.88	1.37, 2.58	<0.001	1.59	1.04, 2.42	0.031
Fully adjusted model: c	Never smoker	1.00	(reference)		1.00	(reference)	
	Former smoker	1.33	1.08, 1.65	0.009	0.88	0.56, 1.39	0.589
	Current smoker	1.80	1.33, 2.45	<0.001	1.75	1.15, 2.67	0.010

Footnotes:

a Odds ratio for *C. difficile* infection (yes/no).

b Incidence rate ratio for number of *C. difficile*-related visits.

c Covariates were year of birth (centered), sex, race, ethnicity, body mass index, alcohol use, marital status, education, total assets, region of residence, heart disease, chronic lung disease, diabetes mellitus, stroke, end-stage renal disease, depression, Crohn’s disease, ulcerative colitis, irritable bowel disease, celiac disease, number of medical visits, number of infection-related visits.

Since the Health and Retirement Study constitutes a multistage probability sample of US households [10], survey-weighting was utilized for the statistical analyses. Survey-weighted rates of CDI were calculated using the number of individuals with CDI as the numerator and person-years of observation as the denominator. Survey-weighted logistic regression was used to assess the association between smoking status and CDI, offset by the log of the person-years under observation. In the fully adjusted model, the following covariates were included: year of birth (centered), sex, race (Caucasian, African-American, other), ethnicity (Mexican-American, other Hispanic [Puerto Rican/Cuban-American/other Hispanic], non-Hispanic), body mass index at first interview, use of alcoholic beverages at first interview, marital status (married or partnered; divorced or separated; single; widowed) at first interview, education (no high school degree; high school degree; education beyond high school), total assets at first interview, region of residence in the US (Northeast, Midwest, South, West) at first interview, heart disease at first interview, chronic lung disease at first interview, diabetes mellitus at first interview, stroke at first interview, end-stage renal disease at first interview, major depression, Crohn’s disease, ulcerative colitis, irritable bowel disease, celiac disease, total number of medical-related visits, and total number of infection-related visits. For those individuals who developed CDI, only covariate information prior to the date of the first recorded CDI diagnoses was used. Predicted marginal probabilities were calculated from the fully-adjusted model taking into account the sampling weights. Population-weighted differences in the probabilities of CDI were assessed comparing former/current smokers with never smokers, with stratification by region of residence.

In addition, we assessed whether smoking was related to the number of CDI-related visits. We initially evaluated the appropriateness of the Poisson model versus zero-inflated Poisson model using the Vuong test [11]. We then used survey-weighted zero-inflated Poisson regression (offset by the log person-years of observation) and included the covariates listed above in the fully adjusted model. Secondary analyses involved the exclusion of individuals with a diagnosis of Crohn’s disease or ulcerative colitis in the regression models. Alpha was set at 0.05, 2-tailed. Statistical analyses were performed by using Stata/MP 11.2 (StataCorp LP, College Station, TX, USA).

Human subjects approval was obtained through the Institutional Review Board at the University of Michigan and the Privacy Board at the Centers for Medicare and Medicaid Services. All analyses were conducted retrospectively on an existing database (http://hrsonline.isr.umich.edu/index.php?p=medicare). Since this was a secondary analysis of existing data, there was no recruitment of subjects for this particular study and therefore, patient consent was not required.

## Results

Among the 16,781 participants in the linked Health and Retirement Study database, 404 individuals were diagnosed with CDI at least once in their records. Overall, the rate of CDI was 220.6 per 100,000 person-years (95% CI: 193.3, 248.0). Of the 16,781 subjects, 19.5% were current smokers and 39.0% were former smokers. Rates of CDI were 281.6/100,000 person-years in current smokers, 229.0/100,000 person-years in former smokers and 189.1/100,000 in never smokers. Table 1 shows strata-specific CDI rates for individual characteristics. Overall, the highest rates of CDI were found among the current smokers. CDI rates were particularly elevated for those individuals with chronic lung disease, stroke, and ulcerative colitis.

Smoking remained statistically significant in a survey-weighted regression model after adjustment for age, gender, and race (Table 2). Smoking was also associated with CDI infection in the fully-adjusted model. The odds of CDI were 33% greater in former smokers and 80% greater in current smokers when compared to never smokers. In addition, there was a significant linear trend in the odds of CDI from never smoking, former smoking to current smoking (p<0.001) in the fully-adjusted model. When the number of CDI-related visits was evaluated (Table 2), only current smokers exhibited a relationship with the number of CDI-related visits. Current smokers had a 75% increased rate of CDI compared to never smokers.

When individuals with Crohn’s disease or ulcerative colitis were excluded from the fully-adjusted models, the odds ratio for former smokers (versus never smokers) was 1.30 (95% CI: 1.01, 1.68; p = 0.044) and for current smokers (versus never smokers) was 1.81 (95% 1.31, 2.51; p = 0.001). The incidence rate ratio for the association between former smokers and number of CDI-related visits was 0.94 (95% CI: 0.61, 1.45; p = 0.785) when persons with Crohn’s disease or ulcerative colitis were excluded. The incidence rate ratio for the association between current smokers and number of CDI-related visits was 1.70 (95% CI: 1.10, 2.62; p = 0.018) when individuals with Crohn’s disease or ulcerative colitis were excluded.

The probabilities of CDI (1991–2007) by smoking status and region of residence are shown in Table 3. CDI was most common in the Northeast, followed by the Midwest, and was lowest in the West. However, within each region of the country, current smokers had the greatest probability of CDI and never smokers had the lowest. The differences in CDI probabilities by smoking status were statistically significant overall, and within each region of the country.

**Table 3:** Probability of *Clostridium difficile* Infection in Medicare Beneficiaries in the United States 1991–2007, by Smoking Status and Region of Residence.

Region	Smoking Status	Probability of CDI a	Difference in CDI Probability b	95% CI for Difference	p value
Northeast:	Never smokers	2.51%	(reference)		
	Former smokers	3.30%	0.79%	0.21%, 1.37%	0.009
	Current smokers	4.40%	1.88%	0.71%, 3.05%	0.002
Midwest:	Never smokers	2.20%	(reference)		
	Former smokers	2.89%	0.69%	0.11%, 1.27%	0.020
	Current smokers	3.85%	1.66%	0.69%, 2.63%	0.001
South:	Never smokers	1.68%	(reference)		
	Former smokers	2.21%	0.53%	0.12%, 0.95%	0.013
	Current smokers	2.96%	1.28%	0.54%, 2.02%	0.001
West:	Never smokers	1.33%	(reference)		
	Former smokers	1.75%	0.43%	0.07%, 0.79%	0.021
	Current smokers	2.35%	1.03%	0.34%, 1.71%	0.004
All Regions:	Never smokers	1.89%	(reference)		
	Former smokers	2.49%	0.60%	0.14%, 1.06%	0.012
	Current smokers	3.33%	1.44%	0.62%, 2.25%	0.001

Footnotes:

a Predicted marginal probabilities from survey-weighted, fully adjusted model. Covariates were year of birth (centered), sex, race, ethnicity, body mass index, alcohol use, marital status, education, total assets, heart disease, chronic lung disease, diabetes mellitus, stroke, end-stage renal disease, depression, Crohn disease, ulcerative colitis, irritable bowel disease, celiac disease, number of medical visits, number of infection-related visits.

b Difference in CDI probability compared to never smokers.

## Discussion

The odds of developing an infection with *C. difficile* were 80% greater in current smokers and 33% greater in former smokers than in never smokers. This suggests that personal habits may influence the development of CDI and could, perhaps, be used to predict infection. In this study, we used a nationally representative sample and included CDI diagnoses that were recorded regardless of whether the individual was hospitalized, visited an emergency room, was a resident at a skilled nursing facility, or visited an outpatient clinic (e.g., physician visits, outpatient surgical visits). There are approximately 37 million aged Medicare fee-for-service beneficiaries in the US, which constitutes the reference population of this study [12]. Such population-based studies are important because the use of hospital databases alone often yields an over-representation of smokers. Individuals who smoke are more likely to be admitted to a hospital or nursing home for multiple reasons (such as pulmonary, cardiovascular, or neoplasm-related diagnoses) and therefore, comparison groups within institutional settings tend to have abnormal distributions for tobacco use. Utilization of the linked databases in our study circumvents this problem by using a nationally representative sample and by including all CDI diagnoses – both community and hospital-acquired. It is notable that a recent report approximated that 75% of CDI cases first occur outside of hospitals in the US [13].

We considered several mechanisms by which this may occur. Since *Clostridium* species are present in cigarettes, the repeated use of cigarettes may serve as an oral portal for the introduction of spores into the gastrointestinal tract. Sapkota and colleagues found that 100% of Camel and Kool Filter Kings cigarettes contained *Clostridium* species, as did 80% of Marlboro Red and Lucky Strike Original Red cigarettes [8]. Not only do unlit cigarettes pose as potential fomites, some bacteria have been shown to survive the smoking process and are viable in the smoked filters [14]. It would be of interest to determine whether *C. difficile* spores, specifically, could survive this process.

It is also possible that smokers are more likely to receive antibiotics, which are strongly associated with CDI [4]. A large longitudinal study indicated that individuals who smoked cigarettes were more likely to receive antibiotics for respiratory tract infections in a dose-response manner [15]. Although we did not have a comprehensive list of medications for the subjects in our study, we adjusted for the number of stays (hospital and skilled nursing facility) and visits (clinic/outpatient, emergency department, and home health) in which any infection was recorded. After adjustment, smoking remained significantly associated with CDI.

Another possible reason for our findings could be that smokers have different gut microbiota than nonsmokers, which might influence the risk for CDI [16], [17]. Prior research has shown that smokers have different nutrient and food intakes compared to former smokers and nonsmokers [18], [19]. It has also been demonstrated that differing food intakes change the composition of the gut flora [20]. While disordered microbial communities in the respiratory tract of smokers have been demonstrated [21], it is conceivable that there are differences in the microbiota within the gastrointestinal tract of smokers as well.

There are limitations to this study, including the use of ICD-9-CM codes to determine CDI. The positive and negative predictive values of the ICD-9-CM code for identifying CDI have been previously reported as 87% and 96%, respectively [22]. In addition, information regarding exposure or infection with *C. difficile* prior to entrance into the Health and Retirement Study was not known.

The overall infection rate in our study was 220.6 individuals with CDI per 100,000 person-years in older Americans from 1991 through 2007. A study of CDI from short-stay hospitals across the United States yielded a similar rate in persons 65 years of age or older (228 diagnoses/100,000 population for 1996–2003), although the rates are not equivalent since we measured people and not diagnoses [23]; it is not uncommon for CDI to relapse within individuals and therefore counting diagnoses will inflate the numerator. Of note, the previous study also indicated higher rates of CDI in the northeastern US and lower rates on the west coast [23], similar to our results. The reason for these regional patterns is not yet known. In retrospect, we attempted to evaluate CDI by year and region to investigate this in more detail, but the rates were too unstable to detect any time-related trends across the four regions.

In conclusion, the rate of infection with *C. difficile* is greater in older individuals who smoke cigarettes than in persons who never smoked. There may be a gradation of effect, as current smokers appeared to be at the greatest risk of CDI when compared to never smokers.
